# Psychiatric risks for worsened mental health after psychedelic use

**DOI:** 10.1177/02698811241232548

**Published:** 2024-03-04

**Authors:** Alessia Marrocu, Hannes Kettner, Brandon Weiss, Richard J Zeifman, David Erritzoe, Robin L Carhart-Harris

**Affiliations:** 1 Centre for Psychedelic Research, Division of Psychiatry, Department of Brain Sciences, Faculty of Medicine, Imperial College London, London, UK; 2 NYU Langone Center for Psychedelic Medicine, NYU Grossman School of Medicine, New York, NY, USA; 3 Carhart-Harris Lab, Departments of Neurology and Psychiatry, University of California San Francisco, San Francisco, CA, USA

**Keywords:** Psychedelics, adverse effects, personality disorder, bottom margin analysis, psychiatric risk

## Abstract

**Background:**

Resurgent psychedelic research has largely supported the safety and efficacy of psychedelic therapy for the treatment of various psychiatric disorders. As psychedelic use and therapy increase in prevalence, so does the importance of understanding associated risks. Cases of prolonged negative psychological responses to psychedelic therapy seem to be rare; however, studies are limited by biases and small sample sizes. The current analytical approach was motivated by the question of whether rare but significant adverse effects have been under-sampled in psychedelic research studies.

**Methods:**

A “bottom margin analysis” approach was taken to focus on negative responders to psychedelic use in a pool of naturalistic, observational prospective studies (*N* = 807). We define “negative response” by a clinically meaningful decline in a generic index of mental health, that is, one standard error from the mean decrease in psychological well-being 4 weeks post-psychedelic use (vs pre-use baseline). We then assessed whether a history of diagnosed mental illness can predict negative responses.

**Results:**

We find that 16% of the cohort falls into the “negative responder” subset. Parsing the sample by self-reported history of psychiatric diagnoses, results revealed a disproportionate prevalence of negative responses among those reporting a prior personality disorder diagnosis (31%). One multivariate regression model indicated a greater than four-fold elevated risk of adverse psychological responses to psychedelics in the personality disorder subsample (*b* = 1.425, *p* < 0.05).

**Conclusion:**

We infer that the presence of a personality disorder may represent an elevated risk for psychedelic use and hypothesize that the importance of psychological support and good therapeutic alliance may be increased in this population.

## Introduction

The resurgence of psychedelics as putative medical interventions (Carhart-Harris and Goodwin, 2017; Sessa, 2018) has yielded evidence in favor of the safety and efficacy of psychedelic therapy as a treatment for a wide range of psychiatric disorders, from major depressive disorder (MDD) to addiction, obsessive-compulsive disorder (OCD), end-of-life anxiety, etc. (Bogenschutz et al., 2015; Carhart-Harris et al., 2021b; Griffiths et al., 2016; Moreno et al., 2006). Moreover, some interest has been reported in psychedelic-assisted therapy for, previously overlooked, personality disorders (PDs; Iliff and Moslehi, 2022; Zeifman and Wagner, 2020). Evidence also exists for post-psychedelic improvements in mental health outcomes in general populations (Mans et al., 2021). This work has inspired an increase in public interest and regulatory changes, including decriminalization in several US states and the development of legal supervised psilocybin use services in the states of Oregon (Oregon, USA; Initiative 34, Measure 109) and similar in Colorado (Colorado, USA; Proposition 122).

Despite evidence supporting psychedelics as catalysts of psychotherapeutic processes, and their low physiological toxicity and risk of dependence (Johnson et al., 2018; Schlag et al., 2022), psychedelic interventions are not risk free, perhaps especially when their use is divorced from psychological support. Case reports have described the occurrence of symptoms resembling psychotic episodes (Cohen and Dilman, 1962; Keeler and Reifler, 1967; Ungerleider et al., 1966), and hallucinogen persisting perception disorder (Cooper, 1955; Frosch et al., 1965; Rosenthal, 1964), which emerged as a Diagnostic and Statistical Manual of Mental Disorders (DSM)-recognized condition in the 1980s (American Psychiatric Association, 1987). Overall, a review of UK experimental and clinical lysergic acid diethylamide (LSD) administration studies up to 1968, totaling 4500 participants and 50,000 LSD sessions, reported 3 cases of completed suicide and 37 cases of prolonged psychoses (Malleson, 1971). Cases of psychological iatrogenesis or serious self-injury after psychedelics are often associated with the use of high or repeated dosages (Erritzoe et al., 2017; Malleson, 1971; McWilliams and Tuttle, 1973; Strassman, 1984), stressful or suboptimal use-contexts (Carbonaro et al., 2016; Schlag et al., 2022), or occurred in individuals with previous or close family history of psychotic disorders (Anastasopoulos and Photiades, 1962; Fink et al., 1966; Hoch et al., 1952; Malleson, 1971; Pauk and Shagass, 1961; Ungerleider et al., 1966).

As of today, few negative and mostly minor adverse effects have been documented in controlled research with psychedelics (Schlag et al., 2022), except nine cases of serious adverse events 3 weeks post-administration of psilocybin in a clinical trial for treatment-resistant depression (Goodwin et al., 2022) and an individual case report (Barber et al., 2022). Until recently, according to one meta-analysis, in more than 2000 patients or participants administered psilocybin, LSD, or ayahuasca in experimental settings, no serious adverse events were reported (dos Santos et al., 2017). Another study suggested that the prevalence of cases of iatrogenesis is less than 0.2% in vulnerable populations (Cohen and Ditman, 1963; Johnson et al., 2018), although it is possible that these rates have been under-sampled or understated.

The apparent low prevalence of long-term negative psychological responses in clinical research is likely due to careful screening and controlled experimental guidelines (Johnson et al., 2008). Of note, modern screening criteria exclude individuals with a relevant psychiatric history of psychosis, borderline personality disorder (BPD), or bipolar disorder from psychedelic trials (Carhart-Harris et al., 2016, 2021b; Johnson et al., 2008). In addition, guidelines include recommendations that the surrounding psychological “set” (i.e., a participant’s psychological preparedness) and environmental “setting” be conducive to a therapeutic response (Carhart-Harris et al., 2018; Haijen et al., 2018; Leary et al., 1963; Studerus et al., 2011, 2012). Thus, with some empirical support, emphasis is placed on the importance of psychological support before, during, and after dosing sessions—helping to ensure positive therapeutic rapport (Gorman et al., 2021; Murphy et al., 2022).

Psychedelics do pose a risk of psychologically unpleasant acute experiences, and subjective responses remain unpredictable (Barrett et al., 2016; Byock, 2018; Russ et al., 2019). Limited sample sizes, biased recruitment and reporting, flawed experimental designs, brief follow-up periods, and imprecise measurement and analysis procedures may be causing rare but significant negative effects to be overlooked or under-reported (Carhart-Harris et al., 2021a; Noorani, 2020).

In this study, we focus on a limited but relevant subset of “worst-case” responders in a relatively large sample of individuals, tracked prospectively, who reported on real-world psychedelic use in a range of environments, from “recreational” to therapeutic settings and ceremonial retreats. We call our approach a “bottom margin” analysis, borrowing a term from behavioral economics (Kahneman, 2011). As opposed to the standard approach of analyzing entire study populations and reporting on group-level summary statistics, such as averages and variance—an approach that sometimes even involves the exclusion of extreme data points (i.e., outlier correction)—bottom-margin or “extreme value” analyses (Leadbetter, 1991), intentionally focus on the “worst cases”—acknowledging their rare prevalence but also exceptional importance.

In the present study, we assess psychometric data on psychological well-being collected via online surveys completed prior to and after an intended use of a psychedelic substance. Data were pooled from three independent studies performed at different times, creating a pool of 806 respondents. We chose to assess whether the risk of adverse psychological responses to psychedelics—defined as cases involving a clinically meaningful worsening in a generic index of mental health (psychological well-being)—could be predicted by self-reported psychiatric history. The motivation for this approach was to identify potentially vulnerable populations, and to ultimately inform risk mitigation messaging and strategies regarding psychedelic treatments for both conduct of academic research and public policy. We hypothesized that the frequency of negative responses would be disproportionately larger in clinical subgroups, such as those commonly excluded from modern trials (e.g., bipolar disorder, psychotic disorder, PD), often because they are considered to be at special risk of clinically iatrogenic responses (Coyle et al., 2018).

## Methods

### Datasets and study design

Datasets analyzed in this study were obtained from three separate, prospective web-based survey studies: Global Psychedelic Surveys 1 and 2 (www.psychedelicsurvey.com) and the Psychedelic Ceremony Study (www.ceremonystudy.com) (Haijen et al., 2018; Kettner et al., 2021). Ethical approval was granted by the Joint Research Compliant Office and the Imperial College Research Ethics Committee. Surveys were implemented through the Alchemer platform (https://app.alchemer.eu/) which ensured participant anonymity and data security per ethical requirements. No financial compensation was offered, and all participants provided written informed consent.

Cohorts were self-selected and comprised of opportunistic volunteer samples recruited via advertisements shared on social media platforms (Twitter and Facebook), psychedelic-related online forums (e.g., Reddit groups), e-mail newsletters (e.g., psychedelicexperience.net), and in case of the ceremony study, direct contact with retreat centers offering psychedelic ceremonies, for survey dissemination. Participant eligibility criteria included the following: at least 18 years old, good comprehension of the English, Spanish or Portuguese language, and having the intention to take a psychedelic drug (e.g., psilocybin/magic mushrooms/truffles, LSD/1P-LSD, ayahuasca, dimethyltriptamine (DMT/5-MeO-DMT), mescaline, or iboga/ibogaine) in the near future/in a retreat setting. As an observational study, participants were not excluded on the basis of current psychiatric status, nor psychedelic use background (naïve or experienced alike). Survey responses were then collected via online platforms: www.global.psychedelicsurvey.com or www.ceremonystudy.com. Although their self-reported data were collected, participants who documented consumption of additional substances alongside psychedelics were ultimately excluded from the final sample.

In these cohorts, a minimum of five surveys were completed by participants at prospective and retrospective time points. All online questionnaires consisted of a large battery of existing and self-constructed measures assessing changes in mood induced by psychedelic use. For full descriptions of individual study methods, see Haijen et al. (2018) and Kettner et al. (2021). For this study, we make use of (a) baseline demographic measurements taken 2 weeks prior to the psychedelic experience, (b) assessments of the subjective psychedelic experience 1 day post-session, and (c) a measure of well-being at baseline (i.e., prior to psychedelic use), 2 weeks, and 4 weeks (key endpoint) post-psychedelic use.

### Outcome measure

In the interest of assessing changes in overall psychological state longitudinally in a nonclinical population (largely with no history of psychiatric disorders), our primary outcome of interest was the Warwick-Edinburgh Mental Well-Being Scale (WEMWBS). The WEMWBS questionnaire assesses psychological well-being via 14 items associated with positive mental health, such as positive affect, satisfying interpersonal relationships, positive functioning, and hedonic and eudaimonic aspects (Tennant et al., 2007). The WEMWBS has been shown to have good content and internal construct validity, as well as satisfactory test–retest reliability (Stewart-Brown et al., 2009), extensively validated in different adult and teenager UK and minority populations (Clarke et al., 2011; Taggart et al., 2013). Various studies have indicated a normal distribution of WEMWBS in the general population; therefore, it can be used in parametric analyses (Ng Fat et al., 2017). Evaluations have demonstrated sensitivity to change in psychiatric populations as well as to external interventions, offering insight into mental well-being across groups (Maheswaran et al., 2012; Stewart-Brown et al., 2011) and being commonly applied by mental health service users and carers (Crawford et al., 2011). Change in WEMWBS was calculated as the difference in individual WEMWBS ratings between our key endpoint (4 weeks post) and baseline (2 weeks prior).

### Statistical analysis

Data from all time points were imported and merged using Matlab (release 2019b) and analyzed via Rstudio (2020).

For the detection of statistically important changes in well-being ratings over time at the intra-individual level, we made use of the standard error of measurement (SEM) of the instrument approach previously used in WEMWBS analyses (Beaton et al., 2001; Maheswaran et al., 2012). Participants were considered to have a meaningful improvement or detriment in their mental well-being according to a decrease (negative responder) or increase (positive responder) on the WEMWBS greater than the 1-SEM threshold at 4 weeks post-psychedelic experience. No response was defined as a nonsignificant change in WEMWBS rating falling −(1 − SEM) ⩽ *x* ⩽ +(1 − SEM). SEM was calculated as follows:

$$\begin{array}{l} \mathrm{Standard}\;{\mathrm{Deviation}}_{\mathrm{Baseline}\;\mathrm{WEMWBS}}\left(\mathrm{SD}\right)\\ \times \sqrt{1}-\mathrm{Reliability}\;\mathrm{Measure} \end{array}$$

As the reliability of the instrument is sample dependent, we calculated the Cronbach α within our population as our measurement of internal consistency of the WEMWBS. According to this calculation, the threshold for significant change was found to be ±2.82 points.

### Regression analyses

Predictions of long-term negative responses to psychedelics, defined as above, were assessed via univariate and multivariate logistic regression models to calculate the odds ratio (OR). The dichotomous-dependent variable encoded the classification of negative response (1) or positive/no response (0). Diagnostic history of PD was used as the main predictor of interest. Additional models included covariates of different psychiatric disorders (psychotic disorder, attention deficit and hyperactivity disorder (ADHD), MDD, bipolar disorder, anxiety disorder, eating disorder, OCD) to assess differential effects and control for potential comorbidities, as well as baseline WEMWBS, to account for potential ‘ceiling’ or “floor” effects. OR was interpreted as a measure of effect size and calculated as ℮^logit regression coefficient^. Statistical significance was set at *p-*value ⩽ 0.05.

## Results

### Demographic Information

The data analyzed here were collected from a total of *N* = 807 participants across all three cohorts, 46.5% (*N* = 375) from the “ceremony study” and 53.5% (*N* = 432) from the “global psychedelic survey” (see “Methods” section). The surveys were completed at baseline by *N* = 2008, at 2 weeks by *N* = 1049 and by *N* = 881 at our key endpoint, after 4 weeks, constituting a 56% attrition or “dropout” rate (Hübner et al., 2020). In our final sample, only participants who reported WEMWBS scores at both baseline and 4 weeks were included (*N* = 807). Participant demographic information collected during the baseline survey is summarized and presented in Table 1. Our final cohort was found to include a majority of individuals with a history of psychedelic use prior to enrolment in the study (94.9%), with only 5.1% naïve to psychedelics.

**Table 1.** Demographic data collected at baseline.

Total	807
Age	38.7 ± 13.7
Gender	807
Male	505 (62.6%)
Female	297 (36.8%)
Other	5 (0.6%)
Nationality	807
US—United States	246 (30.5%)
GB—United Kingdom	189 (23.4%)
CA—Canada	43 (5.3%)
DK—Denmark	35 (4.3%)
DE—Germany	35 (4.3%)
Other	259 (32.1%)
Education	807
None	41 (5.1%)
High school (or equivalent)	164 (20.3%)
Bachelor’s degree (or equivalent)	272 (33.7%)
Post-graduate degree (e.g., Master’s or Doctorate)	330 (40.9%)
Employment	807
Full-time job	417 (51.7%)
Part-time job	118 (14.6%)
Retired	53 (6.6%)
Student	152 (18.8%)
Unemployed	67 (8.3%)
Psychiatric history	807
Ever been diagnosed with at least one psychiatric illness	278 (34.4%)
Never been diagnosed with a psychiatric illness	529 (65.6%)
Previous psychedelic use	801
Never	41 (5.1%)
Only once	199 (24.8%)
2–5 times	138 (17.2%)
6–10 times	141 (17.6%)
11–20 times	96 (12%)
21–50 times	110 (13.7%)
51–100 times	45 (5.6%)
>100 times	31 (3.9%)

Means ± standard deviations and absolute frequencies are shown. Numbers in parenthesis indicate the percentages corresponding to the absolute frequencies.

* At least one of the following psychiatric illnesses: major depressive disorder, bipolar disorder, psychotic disorder, anxiety disorder, substance or alcohol use disorder, hallucinogen persisting perception disorder, attention deficit hyperactivity disorder, obsessive-compulsive disorder or eating disorder.

### Classification of responses to psychedelics

To define the bottom margin of our dataset, we used change scores on the WEMWBS (14–70), a self-rated measure of psychological well-being that assesses well-being using 14 items assessed in relation to the past 14 days. We focused on intra-individual WEMWBS changes between the key endpoint (4 weeks post-use) and baseline (1 week prior to use) crossing the 1-SEM threshold of instrumental error. The Cronbach’s alpha measurement of internal consistency of the WEMWBS was equal to 0.91 in our sample, resulting in an SEM = ±2.82.

According to this calculation, 55.5% (448) of participants were classified as positive responders 28.1% (227) as non-responders, and 16.4% (132) as negative responders in our entire cohort (Table 2). This 16.4% therefore constitutes this study’s operationally defined “bottom margin,” that is, those who showed a clinically meaningful negative psychological response to a psychedelic at 4 weeks. The cohort average WEMWBS score was equivalent to 47 (±9.2) at baseline, 52 (±8.75) at 2 weeks, and 51.6 (±8.86) at 4 weeks. In comparison, the most recent England adult population average on the WEMWBS lies at 49.9 (Fuller and Mindell, 2017). Specifically, the cohort had, on average, an increase in WEMWBS scores of 4.67 points (±8.62) at 2 weeks and 4.56 (±8.16) at 4 weeks relative to baseline.

**Table 2.** Cross tabulation of well-being responses to psychedelics per history of psychiatric diagnosis.

Psychiatric history	Negative responders (%)	Non-responders (%)	Positive responders (%)	*N*
Personality disorder	31.2	18.8	50.0	16
Psychotic disorder	25.0	25.0	50.0	4
ADHD	16.7	22.9	60.4	48
Substance or alcohol use disorder	16.0	40.0	44.0	25
Major depressive disorder	14.6	21.7	63.7	157
Bipolar disorder	12.5	31.2	56.2	16
Anxiety disorder	12.3	21.3	66.5	155
Eating disorder	6.9	31.0	62.1	29
OCD	0.0	25.0	75.0	16
No history of psychiatric illness	18.1	30.2	51.6	529
Any history of psychiatric illness	12.9	24.1	62.9	278
Total	16.4	28.1	55.50	807

Positive responders (WEMWBS change ⩾+2.82); non-responders (−2.82 < WEMWBS change < +2.82); negative response (WEMWBS change ⩽−2.82). Response classification was based on the change in well-being between the endpoint of 4 weeks post-psychedelic experience and baseline as reported on the WEMWBS. Psychiatric history was self-reported as a response to the question: “Do you have a history of diagnosis of: major depressive disorder, anxiety disorder, obsessive-compulsive disorder, bipolar disorder, personality disorder, substance or alcohol use disorder (including alcohol dependence and substance use disorder), psychotic disorder (including schizophrenia and psychosis), eating disorders.” Participants were considered to have no history of psychiatric illness if they had not selected any of the above options or selected the “none of the above” option, and considered to have any history of psychiatric illness if at least one of the above diagnoses was selected.

ADHD: attention deficit-hyperactivity disorder; OCD: obsessive-compulsive disorder; psychotic disorder: psychosis and/or schizophrenia diagnosis.

### Responses to psychedelics stratified by psychiatric diagnostic history

Proportions of negative, positive, and non-responders per psychiatric disorder are presented in Table 2. The highest percentage of negative responders was found for participants with a history of a PD diagnosis (31.2%, *N* = 5), followed by psychotic disorders (25%, *N* = 1). In comparison, the proportion for those with any history of psychiatric illness was 12.9%, and for those with no history of psychiatric illness 18.11%, resulting in a proportion of 16.4% in the total cohort. As bipolar disorder was assessed separately, we suspect these were mostly cases of schizophrenia or related psychotic disorders.

### Trajectories of change in individuals with a history of PD

**Figure 1.**
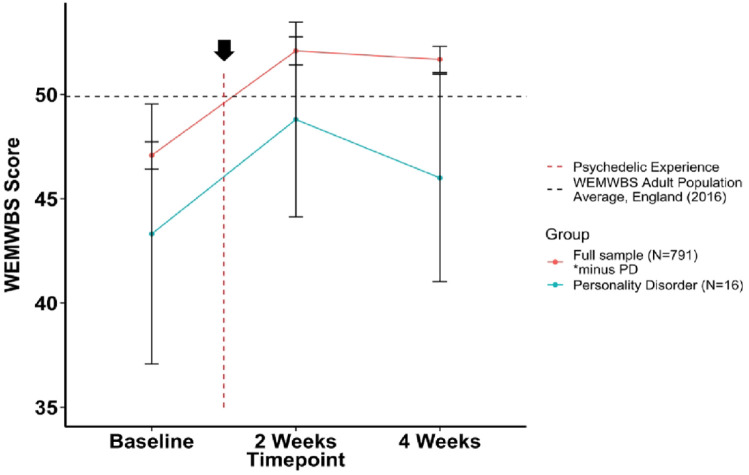
Changes in average WEMWBS score over time in participants with a history of personality disorder versus the rest of the cohort (no history of personality-disorder diagnosis). WEMWBS scores are plotted as group means alongside error bars for standard error of the mean at 2 weeks prior to the psychedelic experience, and 2 and 4 weeks after. Note that the 2 weeks time point refers to 2 weeks after a psychedelic experience, and 4 weeks is 4 weeks after the experience. WEMWBS population average (49.9) refers to the mean WEMWBS score in all adults >16 years old taken from the 2016 Health Survey for England.

As a follow-up analysis, a closer assessment of average changes in WEMWBS scores before and after the psychedelic experience in the entire PD cohort (positive, negative, and non-responders) showed a divergent trajectory compared to the rest of the cohort, as presented in Figure 1. Both the PD subgroup (43.31 ± 12.47) and the overall cohort (47.08 ± 9.12) presented a baseline average WEMWBS score that lay below the national average—that is, WEMWBS = 49.9 (Fuller and Mindell, 2017)—and both showed a significant increase in similar magnitude at 2 weeks post-psychedelic use (+5.67, *p* = 0.01; +4.91, *p* < 0.001), assessed via paired *t*-test. Whereas the majority of the cohort showed a stable positive outcome between 2 and 4 weeks (−0.34, *p* = 0.1), WEMWBS scores in the PD group demonstrated a diverging downward trajectory (−3.6, *p* = 0.08) (Figure 1). Although the mean change (2–4 weeks) between the two groups did not statistically differ, as assessed via a Welch *t*-test (*p* > 0.05), the average WEMWBS scores at the key endpoint were significantly different between groups (*p* = 0.005). Within the PD subgroup, *N* = 5 negative responders were found to significantly decline in WEMWBS compared with baseline (i.e., a decrease of ⩽ −2.82 points). For individual changes in WEMWBS within this subgroup, see Supplemental Figure 1.

### PD diagnosis predicts an elevated risk of iatrogenesis

Based on the changes in WEMWBS observed over time in PD individuals, we investigated whether this diagnostic history may have predictive value on long-term negative outcomes to psychedelic experiences via logistic regression models (Table 3). The binary outcome was coded as a significant negative (1) or positive/no change (0) from the WEMWBS score at baseline. Three models were carried out regressing a dichotomous variable indicating a prior diagnosis of PD (“Personality Disorder”). Model 1 proceeded with a univariate regression, which displayed a positive differential effect of PD on negative psychedelic response, although nonsignificant (0.866, *p* = 0.114, OR = 2.38). In model 2, a multivariate regression including controlling for comorbidities resulted in a larger nonsignificant positive effect (1.208, *p* = 0.065, OR = 3.35). Finally, model 3 added a dependent variable of baseline well-being to control for potential “ceiling” or “floor” effects. In the most complete and justifiable model, therefore, a history of diagnosis of PD was found to confer a 4.16x higher likelihood of experiencing a long-term significant decline in WEMWBS post-psychedelics relative to those with no history of PD (1.425, *p* = 0.043, OR = 4.16). It is important to note that the overall probability of negative responses in the sample was low, as was reflected by the significant negative constant in all of our included models (constant = −4.314, *p* < 0.001). However, we are interested in the marginal effect of PD diagnosis on long-term negative effects relative to participants with no history of PD in our sample. Thus, the greater than four-fold elevated risk must be understood as relative, and with baseline well-being entered as a covariate (as is standard practice). Given the heterogeneity between different PD diagnoses, aimed to understand the profile of participants with self-reported PD, as per a univariate regression on the Ten-Item Personality Inventory (TIPI) elements (Gosling et al., 2003) (Supplemental Table 1). Moreover, in line with our multivariate logistic regression models, we test for correlation between TIPI items and PD diagnosis, conditioning on confounding factors related to baseline well-being and additional psychiatric disorders to identify the underlying characteristics that may predict negative long-term effects on mental health for those with PD (Supplemental Table 2).

**Table 3.** Univariate and multivariate regression models.

Independent variables	Dependent variable: WEMWBS response		
	Model 1	Model 2	Model 3
Constant	−1.65*** (0.1)	−1.5*** (0.11)	−4.31*** (0.63)
Baseline well-being			0.06*** (0.01)
Personality disorder	0.87 (0.55)	1.21^†^ (0.65)	1.43* (0.70)
Psychotic disorder		0.085 (1.26)	−0.61 (1.41)
ADHD		0.02 (0.44)	0.05 (0.45)
Substance or alcohol use disorder		−0.1 (0.6)	−0.35 (0.63)
Major depressive disorder		0.054 (0.3)	0.38 (0.31)
Bipolar disorder		−0.54 (0.85)	−0.80 (0.94)
Anxiety disorder		−0.46 (0.31)	−0.31 (0.32)
Eating disorder		−0.76 (0.75)	−0.66 (0.76)
OCD		−14.8 (594.6)	−14.97 (574.57)

Dependent variable: WEMWBS score encoded as 0: Positive response (WEMWBS change ⩾ +2.82); 1: Negative response (WEMWBS change ⩽ −2.82); Results presented as regression coefficient (standard error).

ADHD: attention deficit-hyperactivity disorder; OCD: obsessive-compulsive disorder.

† *p* < 0.1, *p < 0.05, ***p < 0.001. *p*-Values not corrected for multiple comparisons.

## Discussion

In this study, we made use of real-world data from a naturalistic, observational survey to analyze an operationally defined “bottom margin” of individuals who showed a meaningful worsening of their psychological presentation after use of psychedelics—and then sought to identify psychiatric risk factors predictive of belonging to this category. In a cohort made up of a majority of participants with no reported history of psychiatric illness diagnosis (65.55%), we examined post-psychedelic changes in a generic mental health measure, the WEMWBS, an index of psychological well-being (Tennant et al., 2007). In the total cohort, we identified 16.4% (132 cases) who reported clinically meaningful decreases in well-being at 4 weeks post-experience; these were our “bottom margin” (Kahneman, 2011; Maheswaran et al., 2012). In accordance with the current exclusion criteria of psychedelic clinical trials (Carhart-Harris et al., 2016, 2021b), we found that the highest proportion of negative responders was in a group of individuals with a history of PD diagnosis (31%, i.e., 5 cases of 16, Table 2).

The identification of baseline predictors of psychological iatrogenesis in response to psychedelic experiences is an important yet largely untapped field that has the potential to improve the screening and safety of medical and experimental psychedelic administration. Moreover, despite including a largely healthy cohort with no history of psychiatric disorders, this study provided a unique opportunity to assess responses to psychedelics in groups generally excluded from trials.

Presently, screening criteria in psychedelic therapy trials are based mostly on informal assumptions that are not strongly based on systematic empirical investigations. For example, all protocols include personal and often immediate family history of psychotic illness as exclusion, and some also include PD (e.g., Carhart-Harris et al., 2021b). This, as well as a significant need for better treatments (Alvarez-Tomás et al., 2017; Cristea et al., 2017; Ripoll, 2013), has contributed to some questioning whether even these excluded categories could benefit from psychedelic therapy, perhaps especially so if the treatment was to be tailored to the (presumably) special needs of especially vulnerable populations (Wolf et al., 2023; Zeifman and Wagner, 2020).

As a response to a perceived knowledge gap in the field, potentially related to biased agendas in favor of psychedelics, we chose to selectively focus here on negative responses; however, decisions about clinical research and development should always entail carefully considered cost-versus-benefit calculations. Thus, although the risk of adverse psychological responses did appear to be appreciably elevated in those with self-reported histories of PD in the present study, this does not also imply that they cannot stand to benefit from psychedelic therapy (Müller et al., 2020; Zeifman et al., 2020). Relatedly, readers should recognize that we assessed psychedelic drug use in diverse naturalistic settings; one should not be hasty therefore to make strong extrapolations to clinical contexts or controlled trials with psychedelics.

To gain insight into the type of PD that was described in the PD group within our cohort, we assessed for correlations with individual items in the TIPI (Gosling et al., 2003), measuring the five major personality domains: openness to experience, extraversion, agreeableness, conscientiousness, and emotional stability (inverse of neuroticism) (Costa and McCrae, 2008). We identified a significant negative correlation between emotional stability (i.e., trait “neuroticism” when inverted) and agreeableness items (Supplemental Table 1). This personality structure is considered characteristic of a borderline-like personality disorder profile (Samuel and Widiger, 2008), thus providing some validation for our self-reporting PD-diagnosed subgroup. BPD affects 1.5% of the population (Torgersen et al., 2012; Volkert et al., 2018) and is characterized by behavioral and emotional dysregulation, with difficulties in interpersonal relationships (American Psychiatric Association, 2013). The emotional volatility and instability that characterize BPD could be seen as consistent with an unreliable trajectory of response to psychedelic use—as seen in our PD sample—that is, where improvements in well-being were seen at 2 weeks post-treatment, but marked decreases were seen just 2 weeks later, at the key 4 weeks post-treatment endpoint, although these scores on average did not dip below baseline scores (Figure 1). A similar type of trajectory was identified in recent studies of psychedelic therapy, where participants with BPD diagnosis showed a brief, noticeable reduction in anxiety scores which seemed to return by week 4 post-treatment, with one severe case of acute distress (Anderson et al., 2020; Fineberg et al., 2023). These observations imply a special fragility to short-term responses in individuals with PD and perhaps an elevated need for a more long-term care model.

Psychedelics are known to be psychologically agitative, which is why it is assumed to be essential that they be combined with psychological support to maximize positive response and mitigate risk (Carhart-Harris and Friston, 2019; Grinspoon and Doblin, 2001; Healy, 2021; Koslowski et al., 2022). It is conceivable that the importance of psychological support is even greater in individuals with PD. For example, insecure attachment structures and histories of complex trauma, which are known to be prevalent in BPD (Levy et al., 2005; Yuan et al., 2023), may elevate the risk of insecure therapeutic relationships and phenomena such as “termination reactions”—that is, where the threat of terminating a therapeutic relationship triggers an upsurge in symptomatology (Bender, 2005; Domsalla et al., 2014; Euler et al., 2018; Staebler et al., 2011). Risks of psychological “splitting” (Fertuck et al., 2018; Merced, 2018) or antagonistic actions against psychedelic therapy practitioners, or psychedelic therapy as a paradigm, should also be carefully considered when suspecting the existence of PD in individuals pursuing this treatment. Indeed, a recent randomized pilot trial supporting the safety of psychedelic administration in BPD patients included two participants who, despite initial brief clinical improvements, experienced acute distress and suicidal ideation at week 4, hypothesized to have resulted from study termination—and an emotional reaction to this, that is, a so-called “termination reaction” (Fineberg et al., 2023). Moreover, as the BPD population is characterized by high suicidality and incidences of self-harm (Reichl and Kaess, 2021), there may be an additional layer of risk to treating this already highly vulnerable and psychologically complex population with a psychologically potent and agitating intervention. A recent case report confirms this concern, with intravenous esketamine leading to disinhibited behavior and attenuation of cognitive control, with consequent increased impulsive and suicidal behavior (Vanicek et al., 2022). Contingencies for mitigating such risks may be wise to implement early in the treatment process if indeed treatment is to be considered and attempted in such cases. We note that a leading treatment for BPD is a 12-month psychotherapy (O’Connell and Dowling, 2014). Thus, the brief (e.g., one dose, minimal aftercare) psychedelic therapy package being trialed for treatment-resistant depression (Goodwin et al., 2022) may not be sufficiently safe for cases where there is a diagnosed or suspected BPD.

Despite being excluded from some trials, there have been few studies of psychedelic administration in BPD patients, sparking a debate on the potential effects of psychedelics in this group. The argument for the potential of psychedelic therapy in BPD (Zeifman and Wagner, 2020) may arise, in part, from reported benefits in disorders often comorbid with BPD—for example, MDD, post-traumatic stress disorder, substance abuse (Bogenschutz et al., 2015; Bouso et al., 2008; Carhart-Harris et al., 2021b) including improvements in some key symptoms of BPD (e.g., suicidality, emotion dysregulation, self-image) (Argento et al., 2017; Domínguez-Clavé et al., 2019; Soler et al., 2018; Zeifman et al., 2022). More recently, a small number of pilot studies and case reports have tested this directly, administering ketamine or ayahuasca to patients with BPD, reporting no adverse effects and reductions in suicidal ideation and behavioral symptom severity (Danayan et al., 2023; Fineberg et al., 2023; Nandan et al., 2022; Purohith et al., 2021; Zeifman et al., 2019). Future case studies may help address whether any histories of suspected BPD and related psychological complexity can account for notable complex or adverse responses to classic psychedelic therapy.

A univariate regression model (Table 3) indicated a prior diagnosis of PD as a predictor of long-term negative responses to psychedelics, conferring a 2.38x greater relative risk compared to the rest of the cohort. However, this was nonsignificant, possibly due to a lack of statistical power conferred by our limited sample size (*N* = 16). A subsequent multivariate regression model that better controlled for potential confounders revealed the over four-fold risk of a negative psychological response relative to the remainder of the sample that is reported above. Controlling for baseline well-being was included due to previous psychedelic research studies showing that those with lower-than-average baseline well-being, such as participants with a history of MDD, are more likely to report improvements in mood following psychedelics (Haijen et al., 2018; Mason et al., 2020). Thus, controlling for baseline mental health is a justifiable analytical procedure that can address issues with ceiling and floor effects on measures versus greater scope for change, that is, this being linked to the “regression to the mean” confound (Clifton and Clifton, 2019; Robinson and Jewell, 1991).

In addition, several participants reported a history of more than one psychiatric disorder diagnosis; therefore, we included additional psychiatric disorders in our models to identify unique contributions. In fact, individuals with PDs are known to have high rates of comorbidities with mood disorders, such as MDD and bipolar disorder (Biskin and Paris, 2013; Friborg et al., 2014; Shah and Zanarini, 2018), with often overlapping symptomatology (Henry et al., 2001; Koenigsberg et al., 2002; Ruggero et al., 2010). Controlling for such potential confounds may have contributed to our multivariate regression model identifying a significantly greater than four-fold higher likelihood of experiencing post-psychedelic psychological adverse reactions in those with a formal PD diagnosis. However, it is important to note that the inclusion of additional psychiatric illnesses into the model may have caused a so-called “collider effect” (Supplemental Tables 1 and 2).

It is important to acknowledge the limitations of our study, the main one relating to lower quality of observational data, particularly online self-report data, versus data from controlled research. This study design provided the unique opportunity to gain insight into a sample within which subpopulations presumed to be vulnerable to the effects of psychedelics, and often excluded from research, could be assessed. However, due to their small incidence, our analyses lack statistical power, therefore limiting our ability to draw strong inferences from our findings. It is also important to consider the potential for attrition biases in our data—although see Hübner et al. (2020). Fifty-six percent of our cohort dropped out between baseline and the key 4-week endpoint, and a consistent 50% did so in the PD group. One might speculate that this attrition could have underestimated the relative risk of negative responders, for example, among the self-reporting PD-diagnosed subsample.

Relatedly, the self-reported retrospective aspect of the diagnostic information is a limitation. For example, by asking “Do you have a history of (psychiatric disorder) diagnosis,” participants could state psychiatric comorbidities and/or diagnoses received in the past that no longer apply or were made inaccurately. In addition, the formulation of the questionnaire as it relates to PD did not allow for reports of specific disorder diagnoses, explaining our previous investigations.

It is also important to comment on the special nature of a bottom margin or extreme value analysis. Unlike conventional group averaging, which can mask the relevance of outliers, a bottom-margin approach intentionally selects and focuses on outliers in a population. If viewed through conventional group averaging, readers should note that exactly half of individuals reporting a previous diagnosis of a PD actually experienced clinically meaningful increases in well-being (n = 8 of 16). Thus, our results are specific to a relatively elevated risk of iatrogenesis in those with a history of PD and do not reflect the average or proportionally most likely response in this population (which might be benefit rather than harm). In brief, the strength of the approach we have taken here, that is, in paying careful attention to cases of iatrogenesis, could also be seen as its weakness, for example, in inflating risk versus benefit via a selective focus on the former. Relatedly, for equipoise, future work could be done to examine a “top margin” in a population. In this regard, we note a high proportion of positive responders in participants reporting a history of an OCD diagnosis.

Finally, we acknowledge that WEMWBS is not a clinical scale and future bottom margin-type analyses of psychedelic use data might consider examining clinical rating scales of serious or severe adverse events or specific phenomena. Moreover, third-party confirmation of diagnoses would also improve data quality and the strength of inferences that can be drawn. Future research might seek to further scrutinize preliminary suggestions that psychological iatrogenesis is elevated in individuals with previously diagnosed psychotic disorders, where it may be especially difficult to recruit such cases into studies like this—for example, due to elevated attrition in such cases.

In this study, we find that reported PD in a naturalistic sample is over-represented among individuals who experience negative psychological effects following a psychedelic experience, and discover diagnosis-associated risk. Overall, we present this study as the first observational report of risk associated with PD in the context of psychedelic use and encourage further rigorous investigations to tackle the challenges met here. However, we remind readers to consider our earlier point regarding the importance of factoring the potential for benefit (e.g., 50% of individuals with a PD showed clinically meaningful improvements in well-being) alongside considerations of potential for harm and whether psychedelic therapy could be twinned with more long-term conventional care for PD such as dialectical behavior therapy (DBT) to enhance the risk-to-benefit ratio of either treatment in isolation.

The standard duration of DBT is 12 months and involves weekly individual and group therapy (Linehan, 1993). Such prolonged psychotherapy could mesh well with psychedelic therapy, for example, it could help scaffold against the risk of psychedelic therapy-induced decompensation and complement psychotherapeutic processes catalyzed by psychedelic therapy that require continued support to “work through” (Zeifman and Wagner, 2020). Integrating psychedelic therapy with less resource-intensive versions of BPD treatment (e.g., 6-month DBT, group-only DBT, or Good Psychiatric Management; Gunderson, 2014; McMain et al., 2017, 2022) may also suffice and help to increase accessibility. Relatedly, the practice of psychedelic therapy may require additional training or expertise than currently exists within traditional BPD training. There could be great value in integrating these specializations going forward (e.g., DBT + psychedelic therapy). Such integration will become more salient if psychedelic therapy becomes an approved treatment option on an expanding scale.

To our knowledge, no previous study has directly investigated past histories of specific psychiatric diagnoses as predictors of detrimental psychological responses to psychedelics. Our study therefore addresses an important research gap with a novel approach and its results have timely implications, given that we are on the cusp of major policy changes in relation to access to psychedelic drugs. Especially given that there may be implicit incentives not to assess risk—as it could jeopardize clinical development or potential client pools, we encourage future studies to make use of the type of framework and approach used here, that is, both pertaining to an analysis of “bottom margin” responses and the use of real-world observational datasets. Moreover, prediction of response is arguably one of the most important and fertile future growth areas for psychedelic science and medicine. More specifically, such predictive modeling could enable the field to mitigate risk and adapt treatment approaches in line with personalized medicine ideals. Given the sensitive history and stigma that haunts psychedelic drugs, doing this well may be essential to the sustainability of psychedelic medicine as a paradigm. We hope our study highlights the importance of data collection where experimental policy changes on access to psychedelics are planned, such as in Australia or the State of Oregon.

## Supplemental Material

sj-docx-1-jop-10.1177_02698811241232548 – Supplemental material for Psychiatric risks for worsened mental health after psychedelic use Supplemental material, sj-docx-1-jop-10.1177_02698811241232548 for Psychiatric risks for worsened mental health after psychedelic use by Alessia Marrocu, Hannes Kettner, Brandon Weiss, Richard J Zeifman, David Erritzoe and Robin L Carhart-Harris in Journal of Psychopharmacology
