# Supplementary material for: Psychiatric risks for worsened mental health after psychedelic use
Source: J Psychopharmacol. 2024 Mar 4;38(3):225–35. doi: 10.1177/02698811241232548 (PMC10944581; doi:10.1177/02698811241232548)
Supplement: sj-docx-1-jop-10.1177_02698811241232548 – Supplemental material for Psychiatric risks for worsened mental health after psychedelic use [file sj-docx-1-jop-10.1177_02698811241232548.docx]

**Supplementary Material**

**
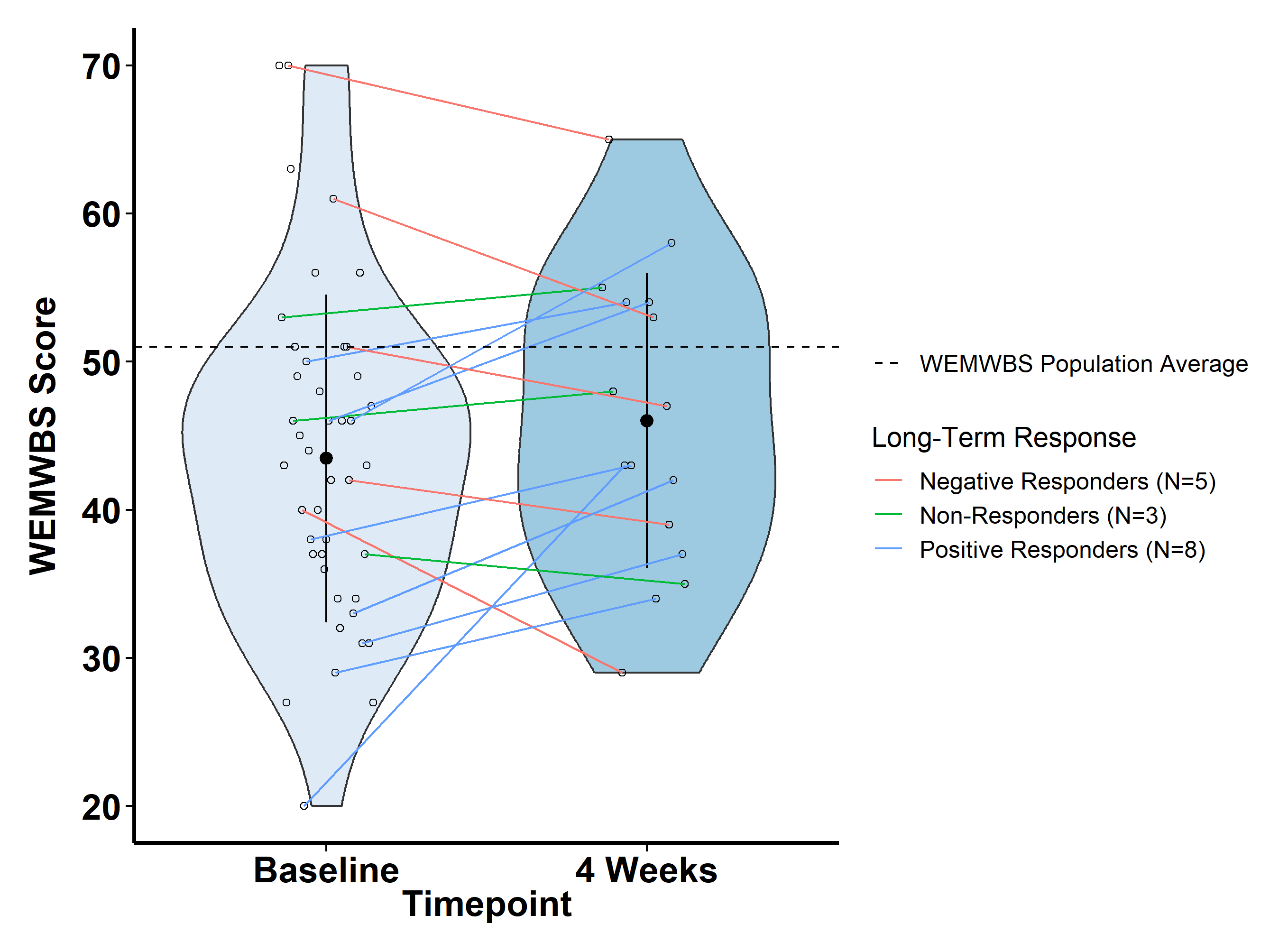
**

**Figure S1. Intra-individual changes in WEMWBS scores over time in participants with a history of personality disorder.** Violin plot with lines indicating separate individuals with prior personality disorder diagnosis (N=16), coloured by their classification of psychedelic response based on WEMWBS change from baseline. Violins display group distributions in WEMWBS scores over time.
*Negative Responders = WEMWBS_baseline_ ≤ -2.82; Non-Responders = -2.82 < WEMWBS_baseline_ < +2.82; Positive responders = WEMWBS_baseline_ ≥ +2.82; NA = Did not report WEMWBS at 4 weeks post-psychedelic experience.*

**Table S1. Linear regression between items on the Ten-Item Personality Inventory (TIPI) in subgroup of individuals with a history of personality disorder diagnosis.**

*Five major personality domains are based on individual items as follows, with R denoting reverse-scoring:*


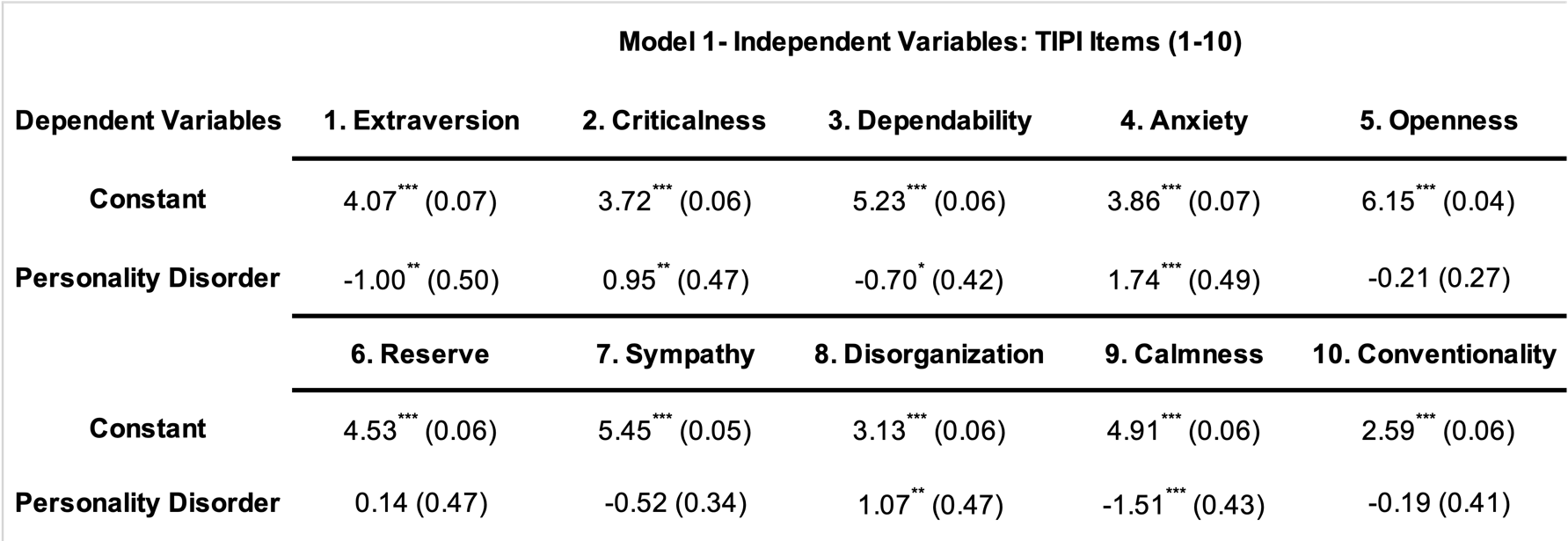
*Extraversion: 1, 6R; Agreeableness: 2R, 7; Conscientiousness: 3, 8R; Emotional Stability: 4R, 9; Openness to Experiences: 5, 10R.*

**Table S2. Multivariate linear regressions between items on the Ten-Item Personality Inventory (TIPI), psychiatric disorders and baseline well-being.**

*Standard coefficients are standardized.*

*Five major personality domains are based on individual items as follows, with R denoting reverse-scoring:*

*Extraversion: 1, 6R; Agreeableness: 2R, 7; Conscientiousness: 3, 8R; Emotional Stability: 4R, 9; Openness to Experiences: 5, 10R.*


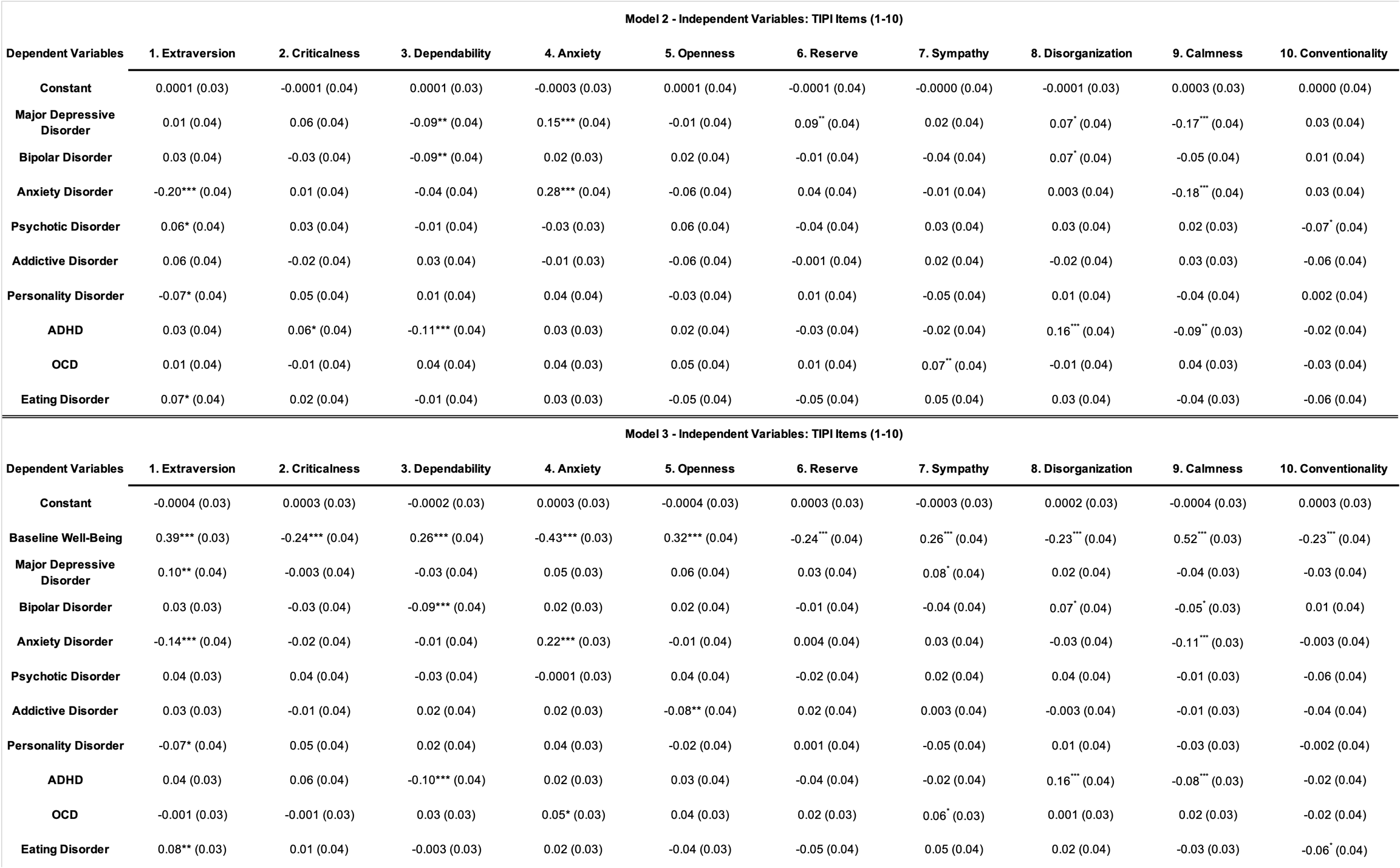
*Extraversion: 1, 6R; Agreeableness: 2R, 7; Conscientiousness: 3, 8R; Emotional Stab*
